# BAROS PROTOCOL IN A UNIVERSITY HOSPITAL: WHAT IS THE IMPORTANCE IN THE POSTOPERATIVE RESULTS OF BARIATRIC SURGERY?

**DOI:** 10.1590/0102-672020230002e1726

**Published:** 2023-03-20

**Authors:** João Evangelista, José Henrique Cardoso Ferreira da Costa, Johnnes Henrique Vieira Silva, Murilo Pimentel Leite Carrijo, Pedro Castor Batista Timóteo da Silva, Daniel Felipe Morais Vasconcelos, e Pedro Cavalcanti de Albuquerque

**Affiliations:** 1 Oswaldo Cruz University Hospital, Bariatric Surgery Service – Recife (PE), Brazil; 2 Universidade de Pernambuco, Faculty of Medical Sciences – Recife (PE), Brazil.

**Keywords:** Bariatric Surgery, Obesity, Preoperative Period, Weight Loss, Cirurgia Bariátrica, Obesidade, Período Pré-Operatório, Perda de Peso

## Abstract

**BACKGROUND:**

Although bariatric surgery is highly effective for the treatment of obesity and its comorbidities, preoperative weight loss has an impact on its results.

**AIMS:**

The aim of this study was to correlate preoperative weight loss with the outcome of bariatric surgery using the Bariatric Analysis and Reporting Outcome System scores.

**METHODS:**

This is a cross-sectional, observational study with 43 patients undergoing bariatric surgery that compared a group of 25 patients with a percentage of preoperative excess weight loss ³8% with a group of 18 patients with a percentage of preoperative excess weight loss <8% or with weight gain. The research took place at the bariatric surgery outpatient clinic of the Oswaldo Cruz University Hospital with patients 1 year after the surgery.

**RESULTS:**

Patients had a mean age of 40.8 years (42.7 percentage of preoperative excess weight loss ≥8% vs. 38.2 percentage of preoperative excess weight loss <8%, p=0.095). No significant difference was found between the two groups regarding preoperative comorbidities and body mass index at entry into the program. Higher preoperative body mass index (48.69 vs. 44.0; p=0.029) was observed in the group with percentage of preoperative excess weight loss <8%. No significant difference was found regarding the percentage of excess weight loss (71.4±15.4%; percentage of preoperative excess weight loss ≥8% vs. 69.47%±14.5 percentage of preoperative excess weight loss <8%; p=0.671), the result of the surgery according to the Bariatric Analysis and Reporting Outcome System scores protocol, the resolution of comorbidities, the quality of life, and the surgical complications between the two groups.

**CONCLUSIONS:**

Based on the available data, it is reasonable that bariatric surgery should not be denied to people who have not achieved pre-established weight loss before surgery.

## RESUMO

**RACIONAL:**

Apesar da cirurgia bariátrica ser altamente eficaz para o tratamento da obesidade e suas comorbidades, ainda não está bem estabelecido o impacto da perda de peso pré-operatória em seus resultados.

**OBJETIVOS:**

Correlacionar a perda de peso pré-operatória com o resultado da cirurgia bariátrica pelos escores do método *Bariatric Analysis and Reportig Outcome System*.

**MÉTODOS:**

Estudo observacional transversal com 43 pacientes submetidos a cirurgia bariátrica que comparou um grupo de 25 pacientes com percentual de perda do excesso de peso pré-operatória igual ou maior a 8% com um grupo de 18 pacientes com percentual de perda do excesso de peso pré-operatória menor a 8% ou com ganho de peso. A pesquisa ocorreu no ambulatório de Cirurgia Bariátrica do Hospital Universitário Oswaldo Cruz com pacientes após um ano da cirurgia.

**RESULTADOS:**

Os pacientes tinham uma média de idade de 40,8 anos (42,7 percentual de perda do excesso de peso pré-operatória ≥8% *vs* 38,2 percentual de perda do excesso de peso pré-operatória <8%, p=0.095). Não foram encontradas diferenças significativas entre os dois grupos em relação às comorbidades pré-operatórias e o IMC na entrada do programa. Foi observado maior IMC pré-operatório (48,69 *vs* 44,0; p=0,029) no grupo com percentual de perda do excesso de peso pré-operatória <8%. Não foram encontradas diferenças significativas em relação ao percentual de perda do excesso de peso (71,4±15,4%; percentual de perda do excesso de peso pré-operatória ≥8% *vs* 69,47±14,5% percentual de perda do excesso de peso pré-operatória <8%, p=0,671), ao resultado da cirurgia pelo protocolo *Bariatric Analysis and Reportig Outcome System*, a resolução das comorbidades, a qualidade de vida e as complicações cirúrgicas entre os dois grupos.

**CONCLUSÕES:**

Com base nos dados disponíveis é condizente que a cirurgia bariátrica não seja negada a pessoas que não atingiram uma perda de peso pré-estabelecida antes da cirurgia.

## INTRODUCTION

Obesity is a chronic disease with different factors for its occurrence and is genetically related to an excessive accumulation of body fat. Excess weight gain causes an increased risk of several diseases, particularly cardiovascular diseases, diabetes, and cancer^11^.

The indication for surgical treatment of morbid obesity is related to the ineffectiveness of clinical treatment and the high risk of mortality from untreated severe obesity^18^. Bariatric surgery is the most effective treatment for obesity, which provides 20–35% of initial body weight loss between 12 and 18 months after surgery^10^.

Preoperative weight loss aims not only to facilitate the surgical procedure but also to transform the patient’s quality in relation to diet with the objective of adapting their eating habits to the postoperative period, correcting vitamin deficiencies, improving insulin resistance, and decreasing obesity-related low-grade systemic inflammation^7,6^.

Some insurance companies in the United States require percent preoperative excess weight loss (PPEWL) in the range of 5–10% before approving surgery. Some bariatric centers successfully prescribe mandatory adherence to weight loss programs before accepting patients for operation^15^. However, potential negative effects associated with a preoperative low-calorie program include patient discomfort, increased costs, treatment denial, increased morbidity in surgery associated with the catabolic state, and a possible delay in the treatment that is required^3,20^.

Oria et al. published the Bariatric Analysis and Reporting Outcome System (BAROS) protocol, which, using a point scale, standardized a set of instruments for evaluating the results obtained with patients undergoing surgery worldwide. This protocol evaluates four main areas, namely, percentage of excess weight loss, changes in medical conditions, quality of life, and postoperative complications^13^. Although there are flaws in the constitution of BAROS, it is still considered a standard method for evaluating bariatric surgery^12^.

In this context, carrying out a survey that correlates preoperative weight loss with the result of bariatric surgery using an instrument standardized worldwide will show if preoperative weight loss will imply not only post-operative weight loss but also the overall result of the surgery.

The objective was to correlate preoperative weight loss with the result of bariatric surgery using the scores of the BAROS method.

## METHODS

This is an observational, cross-sectional, retrospective study with 43 patients undergoing bariatric surgery, which compared a group of 25 patients with a percentage of preoperative excess weight loss ³8% with a group of 18 patients with a percentage of preoperative excess weight loss of <8% or with weight gain. The results of the BAROS protocol were compared in order to correlate preoperative weight loss with the results of bariatric surgery.

The cutoff point of 8% was adopted in relation to the percentage of preoperative excess weight loss based on previous studies and because it is used as a goal in this bariatric surgery service.

The inclusion criteria used in the research were as follows:

- Adults of both sexes, over 18 years of age, who underwent bariatric surgery using the Roux-en-Y gastric bypass technique, who had the surgery 1 year ago, and who signed the Free and Clarified Consent Form previously approved by the Research Ethics Committee of the Hospital of the Oswaldo Cruz University Hospital (nº 3.764.639).

The exclusion criterion in the research was as follows:

- Refusal to participate in the study.

The percentage of preoperative excess weight loss was calculated by subtracting the patient’s weight on the day of surgery from the patient’s weight when he entered the bariatric surgery program, and the result was divided by the patient’s ideal weight loss.

All study participants were monitored by the multidisciplinary team of the Bariatric Surgery Service at Oswaldo Cruz University Hospital, composed of an endocrinologist, nutritionist, psychologist, social assistant, and bariatric surgeon, aiming at significant preoperative weight loss.

Data collection was carried out at the hospital’s outpatient clinic, applying the BAROS protocol, validated in Brazil, which was applied in-person while waiting for the medical consultation 1 year after Roux-en-Y gastric bypass surgery, after the participants signed the Free Consent Form. The data collection period was from January 1, 2020, to September 30, 2021.

**Figure 1:**
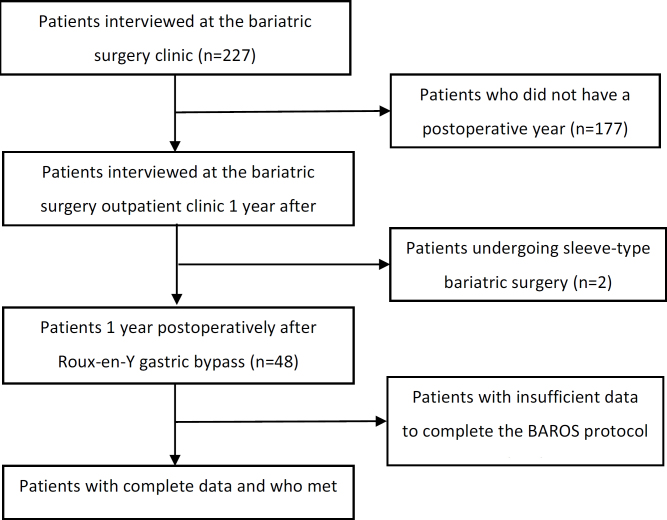
Research protocol.

A total of 227 patients were interviewed, and 177 did not have a year after surgery. Of the remaining 50 patients, 2 underwent sleeve-type bariatric surgery and 5 had insufficient data to complete the BAROS protocol, leaving 43 patients in the research, as shown in Figure 1.

Data were retrieved from the medical records of patients regarding surgical summaries, patient profiles, pre-surgical weight loss, evolution of weight loss after surgery, complications, and changes in comorbidities after gastroplasty.

The BAROS protocol is composed of four domains, namely, percentage of excess weight loss, quality of life, changes in comorbidities, and post-surgical complications. For each domain, a certain score is added, except for the last domain, in which it is reduced from the total score. The answers were evaluated according to a score presented in the table proposed by Oria et al., resulting in a classification with the following possible results: “insufficient,” “acceptable,” “good,” “very good,” and “excellent”^13^.

The diagnosis of hypertension was considered as systolic blood pressure ≥140 mmHg and/or diastolic blood pressure ≥ 90 mmHg, and its resolution as blood pressure below these values only with diet and/or use of diuretics and improvement in blood pressure control with the use of these medications.

The diagnosis of type 2 diabetes mellitus (DM2) was considered as fasting blood glucose above 125 mg/dL, blood glucose above 200 mg/dL 2 h after the oral glucose tolerance test, or glycated hemoglobin above 6.4%. Its resolution was considered when there was normalization of these values only with diet and physical exercise. Its improvement was when there was glycemic control only with oral antidiabetics and without the use of insulin.

The diagnosis of dyslipidemia (DLD) was considered as total cholesterol above 200 mg/dL, HDL cholesterol below 35 mg/dL, and/or triglycerides above 250. Its resolution was considered when there was normalization of these values without the use of medication, and the improvement was when there was normalization of these values with the use of medication.

The diagnosis of osteoarthritis (OA) was considered in the presence of radiological signs and typical symptomatology. Its resolution was considered in the absence of symptoms without the use of medication and its improvement in overcoming the symptoms with the use of medication.

The diagnosis of gastroesophageal reflux disease (GERD) was considered in the presence of typical symptoms with an upper digestive endoscopy ruling out other differential diagnoses. Its resolution was considered in the absence of symptoms without the use of medication and its improvement in overcoming the symptoms with the use of medication.

Data were entered into the Microsoft Excel program, and for all statistical tests, significance was determined at p≤0.05. The descriptive analysis was presented in absolute and percentage frequencies. The paired “t” test was used to compare mean age, body mass index (BMI) at entry into the surgery program, preoperative BMI, BMI after 1 year of surgery, and percentage loss of excess weight. To compare genders, preoperative comorbidities, BAROS protocol results, postoperative complications, quality of life (variable “much better”), and resolution of comorbidities, the chi-square test was used.

## RESULTS

A sample of 43 patients with a mean age of 40.8±10.11 years were interviewed. In total, 76.7% of the participants were women; the mean BMI at the moment they arrived at the University Hospital’s obesity program was 48.76±5.75; mean preoperative BMI was 45.9±6.5; and the mean BMI at the interview moment was 31.3±5.15. The patient characteristics are described in Table 1.

**Table 1:** Patient characteristics and results.

		All patients	≥8%	<8%	p-value
n		43	25	18	
Age, years		40.8±10.1	42.7±10.2	38.2±9.4	0.095
Sex, n (%)					0.895
	Male	10 (23.2)	6 (24)	4 (22.2)	
	Female	33 (76.7)	19 (76)	14 (77.7)	
	BMI – beginning	48.76±5.75	49.3±5.1	47.9±6.5	0.481
	BMI preoperative	45.9±6.5	44.0±5.0	48.69±7.41	0.029
	BMI current	31.3±5.15	30.3±4.18	32.6±6.1	0.161
	%EWL	70.62±14.8	71.4±15.4	69.47±14.5	0.671
Comorbidities (%)					
	HBP	25 (58.1)	15 (60)	10 (55.5)	0.770
	DM2	13 (30.2)	6 (24)	7 (38.8)	0.295
	DLD	13 (30.2)	6 (24)	7 (38.8)	0.295
	GERD	13 (30.2)	6 (24)	7 (38.8)	0.295
	OA	14 (32.5)	9 (36)	5 (27.7)	0.568
Result BAROS (%)					
	Excellent	14 (32.5)	15 (60)	10 (55.5)	0.770
	Great	22 (50.1)	6 (24)	7 (38.8)	0.295
	Good	5 (11.6)	6 (24)	7 (38.8)	0.295
	Fair	2 (4.6)	6 (24)	7 (38.8)	0.295
	Excellent	14 (32.5)	9 (36)	5 (27.7)	0.568
	Great	22 (50.1)	15 (60)	10 (55.5)	0.770
Complications (%)					
	Cholelithiasis	4 (9.3)	3 (12)	1 (5.5)	0.471
	Incisional hernia	8 (18.6)	4 (16)	4 (22.2)	0.602
	Intestinal Obstruction	1 (2.3)	0	1 (5.5)	0.226
	Pneumonia	1 (2.3)	1 (4)	0	0.392
	DVT	1 (2.3)	1 (4)	0	0.392
	Abdominal wall infection	1 (2.3)	1 (4)	0	0.392
	Anemia	9 (20.9)	6 (24)	3 (16.6)	0.2866
	Vitamins deficiency	24 (55.8)	13 (52.2)	11 (61.1)	0.552
	Hair loss	32 (74.4)	17 (68)	15 (83.3)	0.255
Resolution (%)					
	HBP	21 (84)	13 (86.6)	8 (80)	0.656
	DM2	11 (84.6)	6 (100)	5 (71.4)	0.154
	DLD	10 (76.9)	5 (83.3)	5 (71.4)	0.610
	GERD	12 (92.3)	5 (83.3)	7 (100)	0.261
	OA	8 (57.1)	6 (66.6)	2 (40)	0.334

BMI: body mass index; EWL: excess weight loss; HPB: hypertension; DM: diabetes mellitus; DLD: dyslipidemia; GERD: gastroesophageal reflux disease; OA: osteoarthritis; DVT: deep venous thrombosis.

More than a half of patients presented with hypertension (HBP), 30.2% had DM2, 30.2% had DLD, 30.2% had GERD, and 32.5% had OA.

The most common postoperative complications were vitamin deficiency (55.8%) and hair loss (74.4%), followed by anemia (20.9%), incisional hernia (18.6%), and cholelithiasis (9.3%). There was only a single case of intestinal obstruction, pneumonia, deep venous thrombosis (DVT), and abdominal wall infection.

Most of the results evaluated by the BAROS protocol were classified as very good (50.1%), followed by excellent (30.2%) and good (11.6%).

The percentage loss of excess weight was 70.62±14.8%, and there was a high rate of resolution of comorbidities: 84% of arterial hypertension (HBP); 84.6% of DM2; 92.3% of GERD; 76.9% of DLD; and 57.1% of OA.

Most patients considered the quality of life parameters to be much better: self-esteem (88.3%), readiness for physical activity (72%), social relationships (55.8%), work (79%), and interest in sex (55.8%). The quality of life questionnaire results are described in Table 2.

**Table 2:** Life quality.

		All patients	≥8%	<8%	p-value
n		43	25	18	
Self-esteem (%)					
	Much better	38 (88.3)	21 (84)	17 (94)	0.290
	Better	3 (6.97)	3 (12)	0	
	Unaltered	1 (2.32)	0	1 (5.55)	
	Worse	0	0	0	
	Much worse	1 (2.32)	1 (4)	0	
Physical activity willingness (%)					
	Much better	31 (72)	18 (72)	13 (72.2)	0.986
	Better	7 (16.2)	4 (16)	3 (16.6)	
	Unaltered	3 (6.97)	2 (8)	1 (5.55)	
	Worse	2 (4.65)	1 (4)	1 (5.55)	
	Much worse	0	0	0	
Social relationship ability (%)					
	Much better	24 (55.8)	13 (52)	11 (61.1)	0.552
	Better	8 (18.6)	5 (20)	3 (16.6)	
	Unaltered	8 (18.6)	5 (20)	3 (16.6)	
	Worse	3 (6.97)	2 (8)	1 (5.55)	
	Much worse	0	0	0	
Work willingness (%)					
	Much better	34 (79)	18 (72)	16 (88.8)	0.179
	Better	4 (9.3)	3 (12)	1 (5.55)	
	Unaltered	2 (4.65)	2 (8)	0	
	Worse	2 (4.65)	1 (4)	1 (5.55)	
	Much worse	0	0	0	
Sex interest (%)					
	Much better	24 (55.8)	12 (48)	12 (66.6)	0.223
	Better	4 (9.3)	2 (8)	2 (11.1)	
	Unaltered	14 (32.5)	10 (40)	4 (22.2)	
	Worse	1 (2.32)	1 (4)	0	
	Much worse	0	0	0	

Among the 43 patients included in the survey, 58.1% (n=25) had a PPEWL ≥8%, while 41.8% (n=18) did not. Patients had a mean age of 40.8 years (42.7 PPEWL ≥8% vs. 38.2 PPEWL <8%, p=0.095). No significant difference was found in relation to preoperative comorbidities and BMI at entry into the bariatric surgery program (49.3±5.1 PPEWL ≥8% vs. 47.9±6.5 PPEWL <8%; p=0.481).

A higher preoperative BMI (48.69±7.41 vs. 44.0±5.0; p=0.029) was observed in the group with PPEWL <8%.

No significant differences were found in relation to the percentage loss of excess weight (71.4±15.4% – PPEWL ≥8 vs. 69.47±14.5% PPEWL <8%; p=0.671) and the result of surgery by the BAROS protocol, surgical complications, the quality of life questionnaire, and the resolution of comorbidities between the two groups.

## DISCUSSION

Most of the evaluated patients were female (76.7%), according to the findings of several national and international studies^1,15,18^.

The present study demonstrated that 38 (88.3%) of the 43 patients had some comorbidity, demonstrating that obesity is a clinical condition that acts as a risk factor for the onset of other diseases. The most common comorbidities were high blood pressure (HBP) (58.1%), DM2 (30.2%), DLD (30.2%), GERD (30.2%), and OA (32.5%)^15^.

Analyzing the relationship between bariatric surgery and its impact on weight loss, it was noted that, as we have done before, this procedure was proved to be quite effective^14^. The mean BMI before and after surgery was 45.9 and 31.3 kg/m^2^, respectively, which implies a significant reduction in cardiovascular mortality and all-cause mortality associated with excess weight^11^.

After surgery, the patients achieved a significant improvement in relation to their obesity class, going from class III to class I. The mean percentage loss of excess weight was 70.62%, proving that there was success in relation to bariatric surgery, for which the minimum required value is above 50%^4^.

Regarding the evaluation of bariatric surgery results by the BAROS protocol, they were “excellent” in 32.5% of the cases, “very good” in 50.1%, “good” in 11.6%, and “fair” in 4.6% %, consistent with literature data^15^.

Our data did not demonstrate that a PPEWL ≥8% is related to a better outcome of bariatric surgery evaluated by the BAROS protocol, percentage loss of excess weight, post-surgical complications, quality of life, and resolution of comorbidities.

A cohort study published in 2020 with 480,075 patients undergoing bariatric surgery demonstrated that even modest weight loss before bariatric surgery was associated with a lower risk of mortality within 30 days after the procedure. Compared with patients without preoperative weight loss, patients with weight loss greater than 0% to less than 5.0%, 5.0–9.9%, and 10.0% and greater had 24, 31, and 42%, respectively, lower risk of mortality in 30 days^19^.

A study on weight loss before surgery with 20,564 patients undergoing gastric bypass from the Scandinavian Obesity Registry showed that preoperative weight loss was associated with increased weight loss, with the greatest effect seen with BMI >45.7 kg/m^2^ at 1 year postoperatively (OR 2.39; 95%CI 2.10–2.72; p<0.001). In contrast to these findings, a study by Horwitz et al, evaluating compulsory insurance for preoperative weight loss, found no difference between mandatory preoperative weight loss and none at 1 and 2 years postoperatively^5^.

A study published in 2021 that analyzed 2,061 patients who underwent gastric bypass found that patients who achieved 5% preoperative weight loss had similar rates of complications (4.2 vs. 5.1%; p=0.288) and reoperation (3.0 vs. 3.4%; p=0.800) compared to those who lost less or no weight. Patients who achieved 10% preoperative weight loss had increased complications (6.6 vs. 3.7%; p=0.017) and reoperation rates (4.5 vs. 2.7%; p<0.001)^21^.

A meta-analysis published in 2021 of three randomized clinical trials that compared a group with a structured preoperative weight loss regimen compared with standard care found no differences in percentage weight loss between the two groups (SMD 0.007; 95%CI -0.561–0.546; p=0.98). Also, a meta-analysis was performed in this study with prospective and retrospective cohort studies that compared, after 12 months of surgery, a group with preoperative weight loss and another group without preoperative weight loss, which did not show any difference in the percentage of weight loss between the two groups (SMD 0.035; 95%CI -0.163–0.233; p=0.73)^8^.

In 2022, a cohort study was published that analyzed 427 patients undergoing bariatric surgery and observed that greater preoperative weight loss was related to a decrease in hospitalization time (1.8 vs. 1.3 days) but was not associated with a reduction in operative time, overall complication rates, ICU admissions, or intraoperative complications^17^.

The Bariatric Surgery Clinical Practice Guideline (2019), supported by several American Medical Societies, recommends that preoperative weight loss should not be an impediment to performing bariatric surgery, based on conflicting data in the literature regarding the benefit of preoperative weight loss and the potential harm of not having bariatric surgery^9^.

Among this study limitations, it should be underlined the small sample of patients, also it comes to a retrospective study in a single center and without randomization. The analysis performed was only of patients who attended the outpatient consultation, which may have masked the results of the study due to the possible non-inclusion of patients with less satisfactory results. The study analyzed the short-term results of surgery (1 year postoperatively).

## CONCLUSION

The percentage of excess weight loss in the preoperative period ≥8% is not associated with the difference in percentage loss of excess weight in the postoperative period, surgical complications, quality of life, and results of bariatric surgery as evaluated by the protocol BAROS after 1 year of surgery, taking into account various limitations of this study. Therefore, through well-established protocols, it is possible to state that bariatric surgery is not contraindicated for individuals who have not achieved a pre-established weight loss before surgery.
